# Tailoring the Performance of a Composite PEI Nanofiltration Membrane via Incorporating Activated PDA for Efficient Dye Sieving and Salt Separation

**DOI:** 10.3390/membranes15030075

**Published:** 2025-03-02

**Authors:** Wanting Li, Jiaye Liu, Weifu Wang, Shichun Chen, Fengwei Jia, Xiang Li, Ying Zhao, Wenjuan Zhang, Dan Song, Jun Ma

**Affiliations:** 1 Tianjin Key Laboratory of Aquatic Science and Technology, School of Environmental and Municipal Engineering, Tianjin Chengjian University, Tianjin 300384, China; 2 State Key Laboratory of Urban Water Resource and Environment, Harbin Institute of Technology, Harbin 150090, Chinazhaoyinghit@126.com (Y.Z.);; 3 PetroChina Harbin Petrochemical Company, Harbin 150056, China; 4 School of Marine Science and Technology, Harbin Institute of Technology at Weihai, Weihai 264209, China

**Keywords:** activated polydopamine, KMnO_4_ oxidation, polyethylenimine, nanofiltration membranes, dye/salt separation

## Abstract

Efficient dye sieving and salt separation can facilitate the recycling of valuable resources in textile wastewater treatment. This study focuses on developing a high-performance nanofiltration membrane (NF) by co-depositing activated polydopamine (O-PDA), oxidized with KMnO_4_, and polyethyleneimine (PEI) onto a polysulfone support membrane (PSF), thereby enabling effective dye sieving and salt separation. Due to the high hydrophilicity of PDA and the formation of high molecular polymers after oxidation, it was anticipated that O-PDA would crosslink the PEI layer, providing rapid permeating channels. Filtration experiments demonstrated that the formation of O-PDA significantly enhanced the salt retention rate of nanofiltration membranes, achieving a nearly threefold increase in NaCl retention from 15% to 45.7%. It was observed that the retention performance of O-PDA could be adjusted by controlling its loading or oxidation level. Furthermore, despite a notable reduction in permeability, the dye removal efficiency of the O-PDA/PEI membrane increased substantially to 99.5%. Long-term filtration experiments also confirmed both the stability and anti-fouling properties of this membrane design. Clearly, owing to its excellent operational stability and anti-fouling characteristics, the O-PDA/PEI membrane exhibits great potential for applications in dye sieving and salt separation.

## 1. Introduction

In recent years, the rapid development of the printing and dyeing industry has resulted in the frequent occurrence of dye-based pollutants, posing a substantial challenge to environmental management [1,2]. Dye has become one of the most difficult organic contaminants in industrial wastewater treatment [3]. Membrane-based technology has garnered extensive application in industrial wastewater treatment owing to its high removal efficiency and exceptional separation capabilities [4,5]. Among the diverse array of membrane filtration technologies, nanofiltration (NF) stands out as a premier technology with a substantial competitive advantage in industrial wastewater treatment. It excels in efficiently separating salts and organic contaminants [6,7]. However, the adhesion of organic foulants on the membrane surface poses a significant challenge in NF applications, resulting in increased transmembrane pressure, more frequent cleaning requirements, and diminished water quality [8]. A variety of initiatives have been implemented to alleviate membrane fouling. For instance, the application of a hydrophilic layer on the membrane surface via coating or grafting can significantly reduce the interaction between the membrane and foulants by forming a water-rich barrier at the interface between the membrane and the solution [9,10]. Furthermore, minimizing the roughness of the membrane surface can also significantly diminish the attachment of foulants. Consequently, developing a smoother and more hydrophilic membrane surface has emerged as a critical strategy to enhance resistance against organic fouling in NF. In addition, inorganic scaling that primarily resulted from the precipitation of magnesium and calcium compounds, also poses a significant challenge to the effective and efficient operation of membrane-based water purification systems [11,12,13]. The removal of scaling-inducing divalent cations from the source water via nanofiltration can substantially mitigate the risk of scale formation during filtration operations.

Inspired by the remarkable universal adhesion properties of mussels, polydopamine (PDA) has emerged as a promising approach to mitigate membrane fouling and has garnered considerable attention for surface modification [14]. The PDA polymers can be elegantly synthesized through the intricate cyclization and spontaneous polymerization of catechol and amine moieties within dopamine (DA) molecules under alkaline conditions [15]. PDA can securely bond to low-energy surfaces of nonpolar polymers like polytetrafluoroethylene, polystyrene, and polypropylene through electrostatic attractions, hydrogen bonding, and complexation [16,17,18]. Moreover, the catechin segments of PDA have the ability to create covalent linkages with surfaces that have amino functionalities [19,20]. The densely packed PDA coating layer formed on the membrane surfaces functions as an efficient filtration barrier, effectively preventing the passage of small molecules [21,22]. Moreover, the abundance of functional groups within PDA not only significantly amplifies the hydrophilic nature of the membrane surface but also bestows it with exceptional adaptability [23]. This remarkable characteristic allows for versatile customization of the modified membrane surface to cater to specific applications.

Nevertheless, the DA self-polymerization is a time-consuming procedure that typically requires 8 to 12 h or longer to produce a stable PDA layer [23]. The prolonged PDA deposition tends to form large aggregates via noncovalent interactions, leading to a rough surface morphology [24]. These factors limit its applicability in modifying porous membranes. The rapid formation of a structurally uniform and hydrophilic PDA surface layer on the membrane surface remains a critical focus in the field of membrane modification. At present, the incorporation of oxidizing agents such as CuSO_4_/H_2_O_2_, NaIO_4_, (NH_4_)_2_S_2_O_8_, KMnO_4_, and others facilitates the expeditious and homogeneous formation of a PDA coating layer [25]. Although the CuSO_4_/H_2_O_2_ oxidation system has demonstrated consistent and effective catalytic performance, the adhesion of Cu^2+^ ions to the membrane surface impedes its further functionalization [26]. NaIO_4_ oxidation is widely recognized as the most effective among all oxidants; however, its stoichiometric ratio with dopamine is as high as 2:1 [27], significantly increasing the cost of membrane preparation and thereby reducing its feasibility for large-scale surface mixed coatings. The oxidation reaction of (NH_4_)_2_S_2_O_8_ occurs not only at a pH of 4 but can also lead to protein denaturation and the corrosion of copper and other metals [28]. During the oxidation of PDA, the strong oxidizing effect of (NH_4_)_2_S_2_O_8_ may lead to a higher conversion of hydroxyl to carbonyl groups in the structure of PDA, and it may also trigger unwanted side reactions, which may affect the purity and properties of the product [29]. Among these oxidants, KMnO_4_ stands out for its low modification costs, uniform modification, and stability, thereby significantly enhancing the polymerization rate of PDA even at a low molar ratio of KMnO_4_/DA. However, the presence of abundant hydrophobic aromatic moieties in the PDA formed during KMnO_4_ oxidation of DA, results in a limited enhancement of surface hydrophilicity [30].

Polyethylenimine (PEI), a polymer rich in amino groups [31], can efficiently promote the rapid formation of a uniform PDA/PEI layer when incorporated into the KMnO_4_ oxidation of DA process. Nevertheless, the intricate reaction mechanisms and the nuanced alterations in chemical structure on the membrane surfaces during the co-deposition of PDA/PEI in KMnO_4_ oxidation, though intimately tied to the inherent properties of the membrane surfaces, have yet to be fully elucidated. Moreover, the concurrent presence of PEI may engage in a competitive interaction with DA for reactions with KMnO_4_, possibly inhibiting the spontaneous polymerization of PDA and the concurrent deposition of the PDA/PEI blend. Therefore, precisely controlling the amount of KMnO_4_ used in the co-deposition process of PDA and PEI is essential for the efficient development of functional PDA/PEI coatings. While co-deposition coatings of PDA/PEI under KMnO_4_ oxidation have been applied in NF manufacturing, the specific mechanisms of KMnO_4_ during the PDA/PEI co-deposition process and its influence on modulating the intricate and distinctive properties of the membrane surface to enhance fouling resistance warrant further investigation.

The goal of this research was to harness the oxidative power of KMnO_4_ to accelerate the spontaneous polymerization of DA, thereby improving the interfacial bonding between PDA and PEI on the surface of PSf membranes. The surface morphology and composition characteristics of these membranes were examined through various analytical techniques, including scanning electron microscopy (SEM), atomic force microscopy (AFM), X-ray photoelectron spectroscopy (XPS), and attenuated total reflectance Fourier transform infrared (ATR-FTIR). In addition, the wettability and charge changes in membrane surfaces were characterized using a contact angle (CA) goniometer, and a solid-state zeta potential meter. Following this, the membrane permeability and the exclusion capabilities of salts and dyes were assessed for comparative analysis, and the anti-fouling performance of the membranes was also examined.

## 2. Materials and Methods

### 2.1. Membranes and Chemical Reagents

A 20 kDa molecular weight cut-off PSf flat-sheet membrane (Guochu Technology, Xiamen, China) served as the base material for modifications. DA (D&B, Shanghai, China), PEI with 600 Da (Macklin, Shanghai, China), and KMnO_4_ (BR, Tianjin, China) were employed as modification agents. A Tris buffer was created by dissolving trimethylol aminomethane (THAM) (BR, D&B, Shanghai, China) in 30% hydrochloric acid (HCl), (Tianjin, China), and the pH was then adjusted to 8.5. Salts including sodium chloride (NaCl), magnesium chloride (MgCl_2_), sodium sulfate (Na_2_SO_4_), and magnesium sulfate (MgSO_4_) (Tianjin, China) were used for salt rejection experiments. Congo red (CR) (Macklin, Shanghai, China) and methylene blue (MB) (Energy Chemicals, Shanghai, China) were selected for dye rejection studies. Bovine serum albumin (BSA) (BR, Tianjin, China) was used as the model foulant for anti-fouling experiments. Solutions were prepared using deionized (DI) water.

### 2.2. Membrane Modification

**Figure 1:**
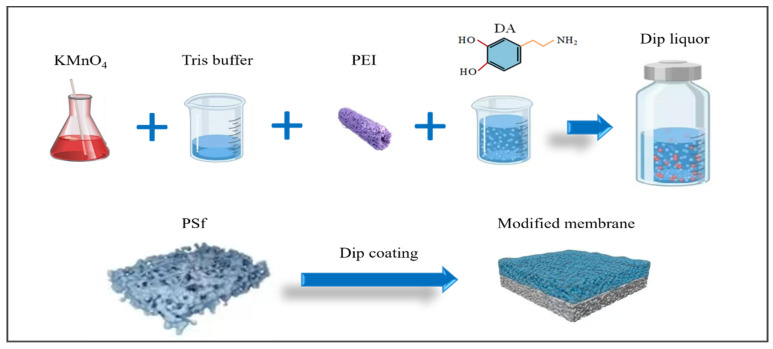
Schematic diagram of the membrane preparation process.

Membrane modification was conducted using the following procedures (Figure 1). (a) The commercially obtained PSf flat membrane was initially submerged in anhydrous ethanol for a duration 2 h, and the residual anhydrous ethanol on the surface of the membrane was cleaned with deionized water. The membrane was then soaked in deionized water and set aside. (b) A measure of 0.666 g/L KMnO_4_ was thoroughly dissolved in the Tris buffer, followed by the sequential addition of varying concentrations of PEI (0, 2, 4, 8, and 12 g/L). A measure of 2 g/L DA was used to prepare various membrane modification solutions. (c)Next, 2 g/L PDA and 2 g/L PEI, without the addition of KMnO_4_, were used to prepare the control membrane (DE). The specific parameters of the modified membranes are detailed in Table 1. (d) The PSf membrane was fitted into a bespoke polypropylene holder. The modification solution, as described in Table 1, was precisely applied to the surface of the membrane for a 1 h coating period. (e) Afterward, the modified membranes were completely washed using deionized water and then dried in an oven at 40 °C over a period of 2 h.

### 2.3. Assessment of Membrane Permeability and Retention Capabilities

**Figure 2:**
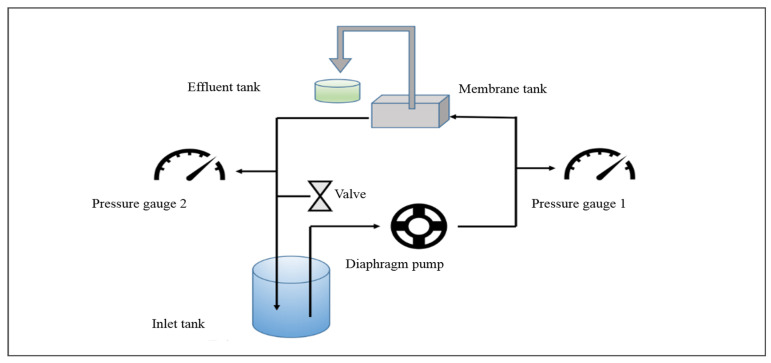
Schematic diagram of the cross-flow membrane filtration test setup.

The characteristics of membrane performance, particularly focusing on water permeability and solute rejection, were rigorously assessed utilizing a bench-scale cross-flow membrane filtration system (Figure 2). To ensure stable permeability performance, the membranes were initially compacted at 0.5 MPa for a minimum of 6 h. Following this, the pressure was decreased to 0.4 MPa to assess the water permeability of the membranes. The cross-flow velocity was maintained 26 cm/s. The effective area of the membrane was 13.7 cm^2^. The experimental temperature was maintained at 25 ± 0.5 °C.

The effectiveness of salt rejection was measured using a conductometric method with NaCl, MgCl_2_, Na_2_SO_4_, and MgSO_4_ as test solutes at a concentration of 500 mg/L. The dye rejection efficiency was evaluated using CR and MB solutions, with molecular weights of 696 Da and 374 Da, respectively. Each solution had a concentration of 100 mg/L. The concentrations of CR and MB in the feed and permeate were quantified using UV-vis spectroscopy at wavelengths of 495 nm and 662 nm, respectively.

The membrane permeability (*J*), as well as the solute or dye rejection rate (*R*), were determined using the following formulas:

(1) $$J\;=\frac{V}{A\;\times \;t}$$

(2) $$R=(1\;-\;\frac{C_{p}}{C_{f}})\;\times \;100\%$$

where *V* represents the cumulative volume of permeate harvested over the time interval *t*, *A* denotes the effective surface area of the membrane, and *C_p_* and *C_f_* correspond to the concentrations of salts or dyes in the permeate and feed solution, respectively.

### 2.4. Organic Fouling Experiments

BSA was employed as a proxy for proteins to assess the membrane fouling behavior. The concentration of BSA was set at 1 g/L and pH was approximately 6.47, and the water permeability of the membranes was measured at 10 min intervals. Ultimately, a one-hour clean utilizing DI water was conducted at the identical pressure to evaluate the restoration of water permeability through the membrane.

The flux recovery ratio (*FRR*) was determined using the following equation:

(3) $$FRR(\%)\;=\frac{J_{R}}{J_{W}}\times 100$$

where *J_W_* represents the initial water permeability and *J_R_* denotes the water permeability following the backwashing process.

### 2.5. Characterization of the Membranes

A UV-vis spectrophotometer (Beijing Purkinje, Beijing, China) was employed to meticulously record the ultraviolet absorbance of various modified solutions at the precise wavelengths of 357 nm and 420 nm. The surface texture and topography of the membranes were meticulously characterized using SEM (Zeiss, Jena, Germany) and AFM (Bruker Dimension Icon, Billerica, MA, USA). The functional groups presented on the membrane surfaces were identified using an ATR-FTIR spectrometer (Nicolet 460, Thermo Fisher Scientific, Waltham, MA, USA). XPS (Thermo Fisher Scientific, Waltham, MA, USA) was used for further analysis of the chemical makeup of the membrane surfaces. The charge changes in the membrane surfaces were further determined using a solid surface zeta potentiometer (Anton Paar, Graz, Austria). The CA of the membrane surfaces was evaluated by the sessile drop method with an automated contact angle measuring instrument (Kruss, Hamburg, Germany).

## 3. Results and Discussion

### 3.1. Optimization of the Co-Deposition Solution

**Figure 3:**
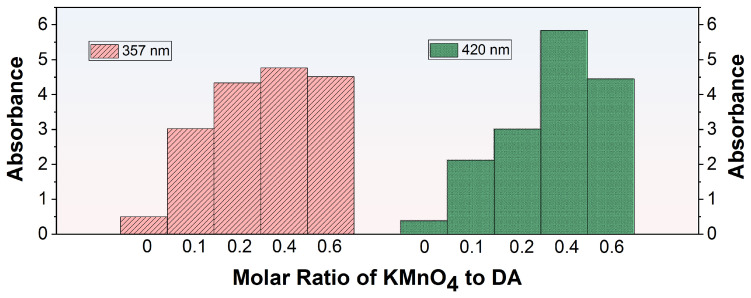
UV-vis absorbance at 357 nm and 420 nm for KMnO_4_-activated DA solutions with varying molar ratios.

In the process of co-depositing PDA and PEI, a functionalized layer was created on the membrane surface through the spontaneous polymerization of DA which was subsequently followed by the conjugation between the resultant PDA and PEI. The introduction of KMnO_4_ can boost the oxidative polymerization of DA, leading to the formation of the activated polydopamine (O-PDA). However, the interaction between KMnO_4_ and PEI may interfere with the formation of the O-PDA/PEI coating. To ensure a consistent and well-adhered O-PDA/PEI layer, it was crucial to identify the optimal concentration of additives before fabricating the modified membranes. UV spectroscopy was utilized to examine the absorbance characteristics of diverse modified solutions, as outlined in Table 1. The absorption maxima at 357 nm and 420 nm in the UV spectra can be attributed to two distinct processes. The peak at 420 nm was due to the self-polymerization of DA, which involved carbonyl groups (C=O). The peak at 357 nm was attributable to the formation of Schiff bases between PDA and PEI, involving imine groups (C=N) [32]. Figure 3 displayed the UV-vis spectra of KMnO_4_-activated DA solutions with different molar ratios at the wavelengths of 357 nm and 420 nm. In the absence of KMnO_4_ oxidation, the UV-vis absorption at 357 nm and 420 nm for the modified solutions was notably low, signifying infrequent occurrences of DA self-polymerization and PDA/PEI co-deposition. A minor increase in the molar ratio of KMnO_4_ to DA marginally improved the PDA/PEI co-deposition, while markedly accelerating DA self-polymerization reaction. Elevating the molar ratio of KMnO_4_ to DA to 0.4 resulted in the peak absorbance at both 357 nm and 420 nm, indicating effective DA oxidation that promoted self-polymerization and the subsequent sophisticated integration of PDA with PEI. Nevertheless, when the molar ratio of KMnO_4_ to DA was raised to 0.6, the absorbance at these wavelengths declined, suggesting that the amino groups in PEI can reduce the oxygen species of excess KMnO_4_ [33], thus suppressing the oxidation of DA and the subsequent co-deposition process. In conclusion, based on the comparison of absorbance at 357 nm and 420 nm, the KDE8-modified solution exhibited the most effective performance.

### 3.2. Analysis of Membrane Surface Properties

#### 3.2.1. Surface Texture and Roughness

**Figure 4:**
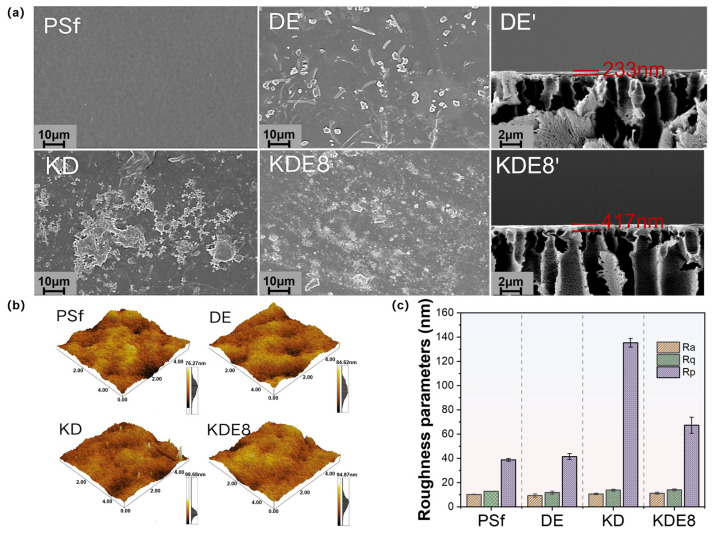
Surface images of the (**a**) PSf, DE, KD, and KDE8 membranes; (**b**) surface roughness of the PSf, DE, KD, and KDE8 membranes; and (**c**) roughness parameters (*R_q_*, *R_a_* and *R_p_*) of the membranes.

The surface texture and roughness of the membranes, in their unmodified and modified states, were examined utilizing SEM and AFM, as depicted in Figure 4. As shown, the PSf membrane displays a relatively smooth surface with clearly visible pores. After the application of the PDA/PEI layer alone, the membrane (DE) displayed an uneven distribution of aggregates and dendrites on its surface, with a modified layer thickness of approximately 233 nm. A significant quantity of polymers was observed on the surface of the membrane (KD) coated with the KMnO_4_-activated PDA layer, exhibiting pronounced agglomeration and non-uniform distribution. It was noteworthy that in the co-deposition-modified membrane (KDE8) of PDA and PEI activated by KMnO_4_, a significantly higher number of polymers were attached to the surface, resulting in a more uniform distribution (Figure 4a). The findings confirmed that KMnO_4_ significantly enhanced the polymerization rate of PDA, and the introduction of PEI cross-linking improved the uniformity of the coating. The modified layer measured approximately 417 nm, which was notably thicker than that of the DE membrane. This observation further substantiated that KMnO_4_ promoted greater polymer attachment to the membrane surface.

The AFM imaging and roughness data of the membrane surfaces before and after deposition are shown in Figure 4b,c. It was evident that the surface morphologies of both the PSF-based membrane and the DE membrane were similar; however, the surface roughness of the DE membrane (*R_a_* = 9.33 nm, *R_q_* = 11.78 nm) was slightly lower than that of the PSF-based membrane (*R_a_* = 10.22 nm, *R_q_* = 12.87 nm). This reduction in roughness can likely be attributed to the slower deposition process of the PDA/PEI layer, which resulted in a more uniform coating, thereby decreasing the surface roughness. The *R_p_* value for the KD membrane surface was notably greater than that of the PSf and DE membranes, primarily due to the hydroxyl radicals produced by the KMnO_4_ oxidation, which boosted the rate of DA aggregation. This led to the creation of larger particles via non-covalent interactions throughout the deposition procedure [34,35]. Nonetheless, following the incorporation of PEI, the *R_p_* value was reduced to 67.37 nm, indicating a more even distribution of the O-PDA/PEI layer across the surface of the base membrane. This could be due to the interference with the non-covalent interactions among PDA polymers as a result of adding PEI [36]. Additionally, the *R_a_* and *R_q_* values for the KDE8 membrane exhibited minimal changes in comparison to the DE membrane, which further suggested that the co-deposition of PDA/PEI triggered by KMnO_4_ was successful in preserving the surface evenness of the membrane.

#### 3.2.2. Chemical Compositions of Membrane Surfaces

**Figure 5:**
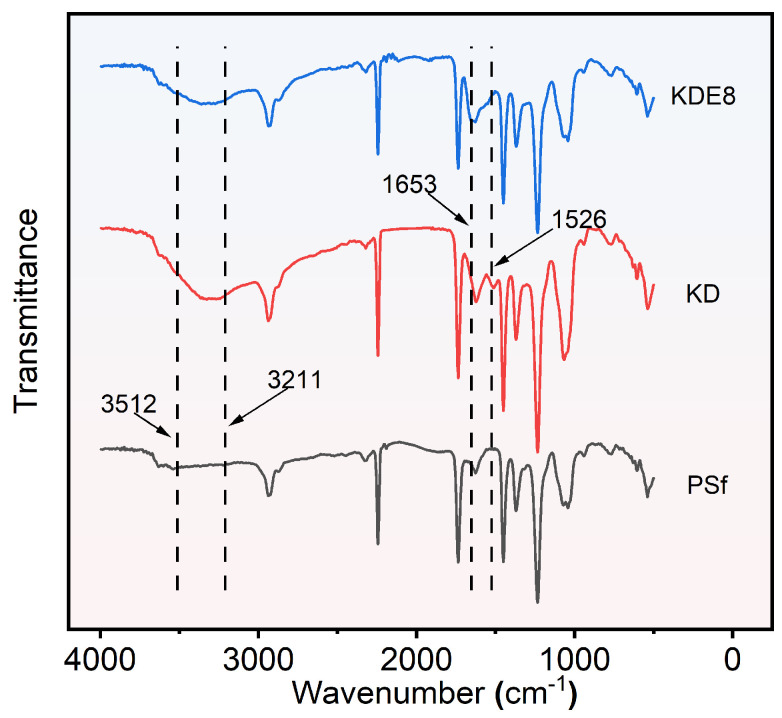
ATR-FTIR spectra of the PSf, KD, and KDE8 membranes.

The FTIR spectra for the PSf, KD, and KDE8 membranes are shown in Figure 5. In contrast to the PSf base membrane, the KD and KDE8 membranes exhibited a stronger characteristic absorption band in the range of 3211–3512 cm^−1^, which was indicative of the stretching vibrations of hydrogen bonds associated with -OH and -NH groups. This observation suggested the effective deposition of DA onto the surfaces of the PSf membrane [37,38]. Upon the application of the PDA/PEI coating, an intriguing novel absorption peak emerged at 1526 cm^−1^ on both the KD and KDE8 membrane surfaces. This distinctive peak was attributed to the characteristic N–H vibrations inherent in both PDA and PEI [39]. After treatment with KMnO_4_, the KDE8 membrane displayed a marked increase in the infrared absorption peak at 1653 cm^−1^, signifying a significant stretching vibration of the C–N double bond. This evidence supported the formation of Schiff base linkages between the amino groups from PEI and the quinone derivatives generated from the oxidation of DA [10,40]. Consequently, the effective formation of Schiff base reaction products of PDA and PEI on the PSf membrane surface had been conclusively confirmed.

**Figure 6:**
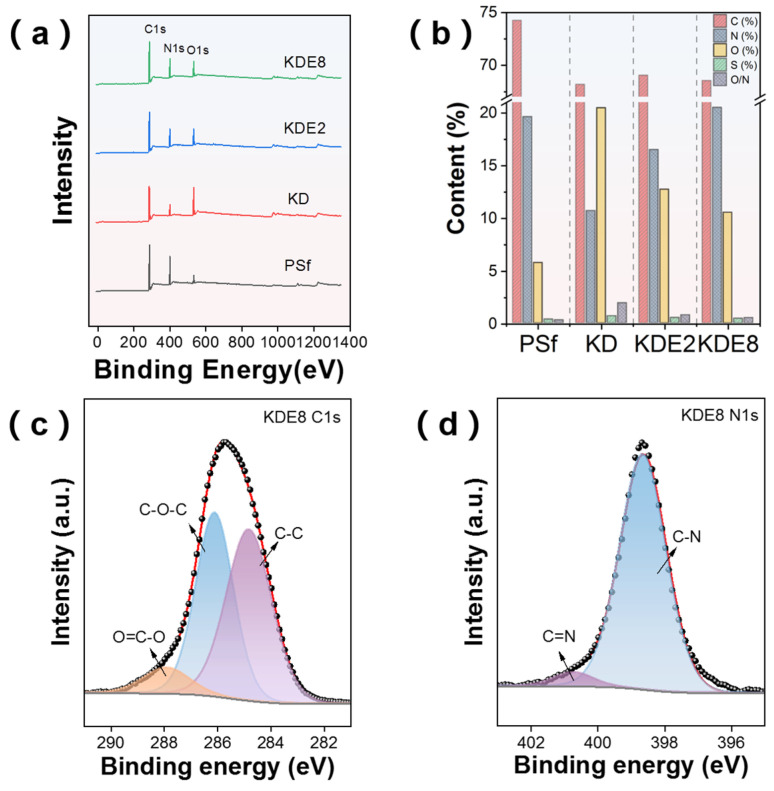
(**a**) XPS spectra overview for the PSf, KD, KDE2, and KDE8 membranes. (**b**) Elemental distribution on the membrane surfaces pre- and post-modification. High-resolution XPS N1s spectra of the (**c**) KDE8-C1s and (**d**) KDE8-N1s.

XPS offered detailed chemical information about the membrane surfaces before and after modification, as illustrated in Figure 6. The analysis revealed that the PSf, KD, KDE2, and KDE8 membranes shared comparable characteristic signals for the C 1s, N 1s, and O 1s orbitals (Figure 6a). Nevertheless, the addition of PEI resulted in a heightened intensity of the N 1s peak and a diminished intensity of the O 1s peak, which was associated with the unique chemical constituents of PDA and PEI. Following the deposition of PDA onto the PSf membrane surface, the atomic percentages of C and O rose to 20.47% and 10.68%, 12.72% and 16.49%, and 10.52% and 20.50% for the KD, KDE2, and KDE8 membrane surfaces, respectively (Figure 6b). In these cases, the reduced O/N ratio observed in the KDE2 and KDE8 membranes provided compelling evidence of the successful surface modification with PEI [41]. Moreover, this surge was also linked to the incorporation of amino groups and hydroxyl from the catechin segments of PDA. The C1s and N1s peaks of the KDE8 membranes were deconvoluted, as shown in Figure 6c,d. In the C1s spectra, three distinct peaks were identified: C-C, O-C-O, and O=C-O. Notably, the O=C-O peak primarily originates from PDA [34]. Meanwhile, the N1s spectrum exhibited two prominent peaks corresponding to C-N and C=N groups. Specifically, the C=N peak is attributed to the formation of a Schiff base structure through the reaction between the quinone group derived from DA and the amino group of PEI [42].

#### 3.2.3. Surface Wettability and Charge

**Figure 7:**
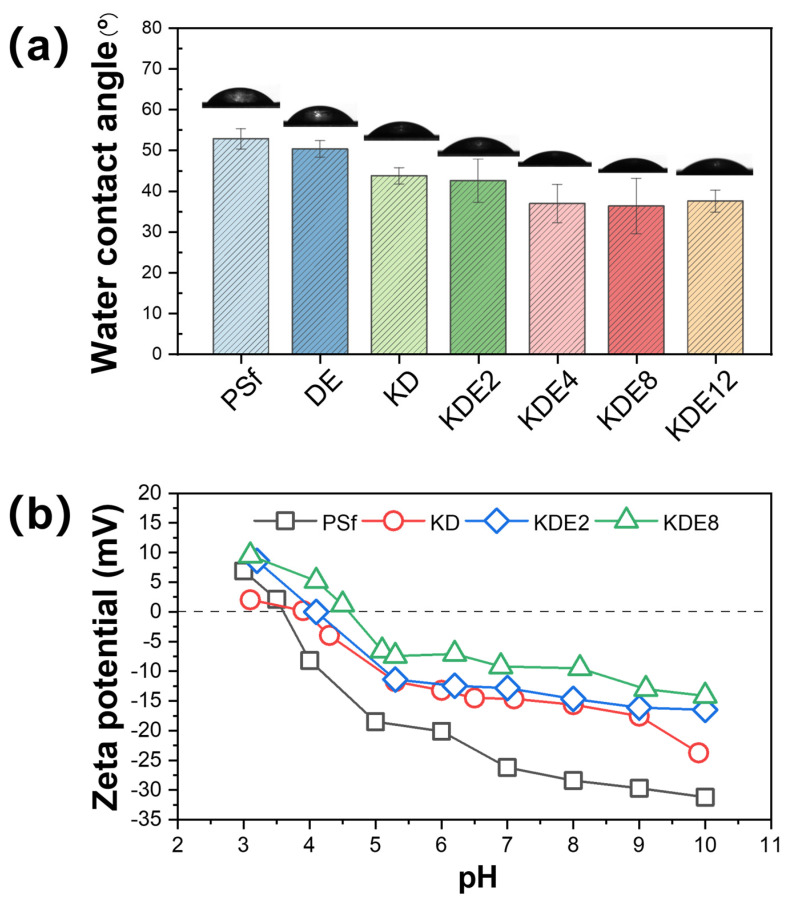
The changes in (**a**) CA and (**b**) zeta potential on the membrane surface pre- and post-modification.

The wettability of the membrane surface played a pivotal role in the progression of membrane fouling. A surface exhibiting hydrophilic properties tended to form a water layer at the interface between the membrane and the solution. This water layer functioned as a barrier, reducing direct contact between the membrane and foulants through spatial hindrance, thereby enhancing the membrane’s fouling resistance [43,44]. Consequently, the CA of the membranes were assessed both prior to and after modification to gauge the hydrophilic nature of their surfaces, as shown in Figure 7a. The PSf membrane displayed an initial CA of 52.8°. After the formation of the PDA/PEI layer on the DE membrane, the CA decreased to 50.3°. This reduction in CA can be credited to the incorporation of hydrophilic amine and hydroxyl groups from DA, which in turn increased the membrane’s hydrophilic nature [45,46]. Nonetheless, when activated with PDA using KMnO_4_ exclusively, the CA of the KD membrane decreased even more, reaching 43.7°. This reduction was presumably due to the enhanced occurrence of hydroxyl and amine groups, which was a consequence of the intensified self-polymerization of DA. Additionally, the incorporation of PEI polymers consistently enhanced the hydrophilicity of the membrane surface (KDE2, KDE4, KDE8, and KDE12) due to the abundance of hydrophilic amine groups [47,48]. Therefore, increasing the concentration of PEI from 2 g/L to 12 g/L significantly reduced the membrane water contact angle from 42.5° to 36.3°. Moreover, an overly high concentration of PEI can impede the polymerization of DA and deposition of formed PAD [49]. In summary, the O-PDA/PEI membrane treated with 8 g/L PEI (KDE8), displayed the minimum CA and the greatest hydrophilicity on its surface. This finding corresponded with the peak UV-vis absorbance noted in the associated modification solution, confirming the optimal hydrophilic enhancement achieved with this concentration of PEI.

The zeta potential changes in the membrane surfaces were evaluated both before and after modification across a pH range from 3 to 10 using streaming potential measurements (Figure 7b). As evidenced, the membrane surface charge decreased with increasing solution pH owing to the ionization of functional groups within the membranes [50,51]. The isoelectric point of the PSf membrane was determined to be 3.60. At a pH of 8.0, the PSf membrane carried a negative charge, facilitating the accumulation of the PDA/PEI blend on its surface. After the deposition of PDA, the zeta potential of the KD membrane increased from −28.40 mV to −15.62 mV due to the introduction of amino groups from PDA [52]. PDA can ionize hydroxyl and amino groups in water with both positive and negative charges, and the isoelectric point of the membrane surface was increased to 4.10. The zeta potentials of the KDE2 and KDE8 membranes increased to −14.65 mV and −9.51 mV, respectively, primarily due to the incorporation of a large number of amino groups in PEI [53]. Thus, the isoelectric point of the membrane surface is further increased to 4.60.

### 3.3. Membrane Filtration Efficacy

**Figure 8:**
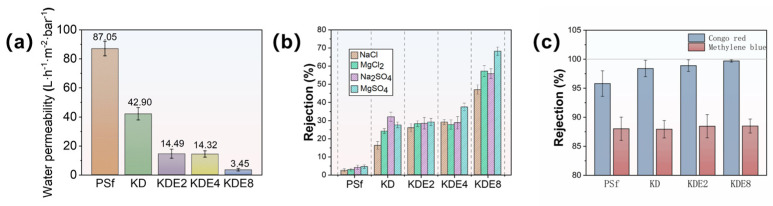
(**a**) Membrane permeability, (**b**) rejection of salts, and (**c**) dyes of pre- and post-modification.

The cross-flow NF setup depicted in Figure 2 was utilized to assess the permeability and separation efficiency of the membranes, with the results presented in Figure 8. The water permeability of the PSf membrane was 87.05 L/m^2^·h·bar. The permeability through the KD, KDE2, KDE4, and KDE8 membranes was markedly diminished following the deposition of PDA and PDA/PEI layers, largely attributable to the formation of aggregate pore resistance on the surfaces of the treated membranes. This phenomenon resulted in a decrease in the pore dimensions of the membranes, as depicted in Figure 8a. In comparison to the KD membrane, the KDE membranes (O-PDA/PEI membranes) exhibited lower water permeability, likely due to the denser PDA self-polymeric layer formed on the surface as a result of KMnO_4_ oxidation. The hydroxyl radicals produced by the activation of KMnO_4_ can enhance the co-deposition rate of PDA/PEI. Furthermore, there was a direct relationship between the concentration of hydroxyl radicals and the thickness of the coating layer on the membrane surface. As the coating density increased, it led to a higher diffusion barrier for water, substantially decreasing the water flux of the KDE8 membrane to a value of 3.45 L/m^2^·h·bar. Compared to the commercial NF-270 membrane, the KDE8 membrane demonstrates lower water permeability (7.67 L/m^2^·h·bar) [54]. Future research should concentrate on optimizing this characteristic to improve its overall performance.

The effectiveness of the membranes in filtration, both in their original and modified states, was evaluated using solutions containing a diverse array of cations and anions, such as NaCl, MgCl_2_, Na_2_SO_4_, and MgSO_4_, as depicted in Figure 8b. The layering of PDA and O-PDA/PEI on the membranes resulted in a contraction of the pore sizes, which consequently elevated the rejection percentages for NaCl in the KD (to 16.20%), KDE2 (to 25.90%), KDE4 (to 29.10%), and KDE8 (to 47.0%) membranes. The pH of all four salt solutions was close to neutral. At this pH, the KDE8 membrane exhibited the weakest negative charge, leading to minimal electrostatic repulsion between the SO_4_^2−^ ions and the membrane surface. However, the co-deposition of the O-PDA/PEI layer resulted in the dense mulch shrinking the pores, thereby enhancing the rejection rate of Na_2_SO_4_ (55.8%). The KDE8 membrane, characterized by a reduced negative surface charge, demonstrated a notably superior rejection rate for MgCl_2_ (57.2%) compared to the other membrane variants. Therefore, for the O-PDA/PEI (KDE8) membrane, the salt rejection order was modified to MgSO_4_ (68.20%) > MgCl_2_ (57.20%) > Na_2_SO_4_ (55.80%) > NaCl (47.0%). The inorganic salt rejection rates of the NF-270 membrane followed the order: Na_2_SO_4_ (98.06%) > MgSO_4_ (96.02%) > MgCl_2_ (42.20%) > NaCl (20.11%) [54]. Notably, the KDE8 membrane demonstrated significantly higher retention rates for MgCl_2_ and NaCl compared to commercial membranes, indicating its superior performance in chloride ion screening. The diverse efficiency observed in salt rejection were linked to the differing surface charge attributes of the various membranes. In particular, the O-PDA/PEI membrane, characterized by a reduction in negative surface charge, showed enhanced electrostatic repulsion (Donnan exclusion) towards divalent cations such as Mg^2+^. These findings implied that the O-PDA/PEI membrane had significant potential for the targeted extraction and recovery of divalent cations from wastewater.

Furthermore, the removal efficiency of organic contaminants by the membranes, both before and after modification, was evaluated through the rejection of Congo red and methylene blue (Figure 8c). The O-PDA/PEI membranes (KDE2, KDE4, and KDE8) demonstrated significantly higher rejection rates for Congo red compared to the PSf membrane, whereas there was no notable difference in the removal efficiency of methylene blue. Specifically, the KDE8 membrane exhibited the highest rejection rate of Congo red at 99.7%, whereas its rejection of methylene blue (88.49%) was similar to that of the other membranes. This can be attributed to the more densely packed active layer of KDE8 membrane. In addition, the molecular weight of methylene blue (319.9 Da) was significantly smaller than that of Congo red (696.7 Da). Consequently, size exclusion emerged as the predominant rejection mechanism for dye removal by the O-PDA/PEI modified membrane. The O-PDA/PEI membranes demonstrated markedly superior rejection rates for Congo red and salts when contrasted with the PSf membrane, signifying an improved selectivity for dye and salt separation in the treated membranes. The removal capacity of the O-PDA/PEI membrane for dyes was comparable to that of the NF-270 membrane, suggesting that the modified membrane could serve as a viable alternative to the NF-270 membrane.

### 3.4. Anti-Fouling Performance of the Membranes

**Figure 9:**
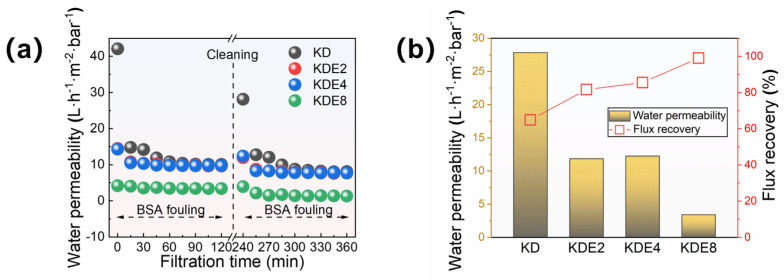
(**a**) Membrane permeability in two cycles of filtration and (**b**) the flux recovery of these membranes after BSA fouling cleaning processes.

The anti-fouling properties of the membranes were further assessed through foulant experiments, using BSA as a model. The membranes’ permeation capabilities were examined both before and after the onset of fouling, and the efficiency of their recovery was later measured following backwashing procedures (Figure 9). Initially, the KD, KDE2, KDE4, and KDE8 membranes exhibited distinct water permeation rates. However, these rates aligned to comparable levels post-fouling with BSA, save for the KDE8 membrane. The O-PDA/PEI coated membranes (KDE2, KDE4, and KDE8) demonstrated greater resilience to organic contaminants than the O-PDA coated membrane (KD), indicating an improved resistance to organic fouling.

The permeability of the O-PDA/PEI membrane can be substantially restored through cleaning process, yielding an impressive recovery rate of water permeability for BSA contaminants at 99.04%. Compared to the NF-270 membrane, which exhibited an 82.0% flux recovery, the modified membrane demonstrated significantly superior flux recovery and enhanced resistance to fouling. This phenomenon can be attributed to the reduced flux and smaller pore size of the modified membrane, which enhanced its capability to trap pollutants on the surface rather than within the pores [55]. As a result, physical cleaning became more effective, leading to an increased FRR value. Conversely, the BSA-fouled O-PAD membrane, which lacked the incorporation of PEI, only achieved a recovery rate of less than 65%. Evidently, the O-PDA/PEI membrane, by developing a uniformly dense structure, effectively prevented protein penetration into the membrane pores, thereby significantly reducing fouling obstruction. Additionally, the O-PDA/PEI membrane presented a more electroneutral surface, which effectively reduced electrostatic interactions with charged particles, thereby increasing its resistance to fouling. Moreover, the hydrophilic characteristics of the membrane’s surface played a crucial role in providing steric hindrance against foulants. This was because the hydrophilic membrane surface facilitated increased contact and adsorption of water molecules, forming a hydration layer that reduced direct contact between pollutants and the membrane [56]. Consequently, this mechanism collaboratively enhanced the anti-fouling capabilities of the O-PDA/PEI membrane.

## 4. Conclusions

In this work, an O-PDA/PEI NF membrane was prepared by accelerating the deposition rate using KMnO_4_ oxidation, which improved its ability to resist organic foulants and enhanced its desalting ability. The hydroxyl radical produced during the oxidation of KMnO_4_ promoted the self-polymerization of DA. However, the introduction of PEI competed with KMnO_4_ for the reaction site, thus inhibiting the formation of the PDA complex. The optimum stoichiometric proportions of the constituents in the altered solution were ascertained using UV-vis spectrometry. The membranes fabricated using 0.666 g/L KMnO_4_, 2 g/L DA, and 2 g/L PEI as optimal components exhibited more even and less textured surfaces, thus improving hydrophilic properties, and increased surface neutrality. Owing to the greater electrical neutrality and more compact surface layer of the O-PDA/PEI membranes, the removal efficiency of solutes, including salts and dyes, was significantly enhanced. In addition, the O-PDA/PEI membrane supported good selectivity for the separation of bivalent ions and Congo red. The O-PDA/PEI membrane exhibited remarkable anti-fouling performance, achieving a maximum BSA permeability with a recovery rate of 99.04%. This superior performance can be attributed to the significant enhancement in surface hydrophilicity and electroneutrality. It was evident that incorporating KMnO_4_ oxidation into the PDA/PEI co-deposition process sped up the oxidation of DA and boosted the quantity of reactive sites for the Schiff base reaction, thereby facilitating the formation of an electroneutral film surface. Conclusively, the membrane formed by the co-deposition of PDA and PEI by oxidation with KMnO_4_ exhibited substantial potential for targeted salt extraction and the reclaiming of resources from wastewater.

## Figures and Tables

**Table 1:** Surface modification parameters corresponding to the assigned membranes.

Membranes	KMnO_4_	PEI	DA	Deposition Time
	(g/L)	(g/L)	(g/L)	(h)
DE	-	2	2	1
KD	0.666	0	2	1
KDE2	0.666	2	2	1
KDE4	0.666	4	2	1
KDE8	0.666	8	2	1
KDE12	0.666	12	2	1

## Data Availability

The raw data supporting the conclusions of this article will be made available by the authors on request.

## References

[1] Maurer L., Villette C., Reiminger N., Jurado X., Laurent J., Nuel M., Mosé R., Wanko A., Heintz D. (2021). Distribution and degradation trend of micropollutants in a surface flow treatment wetland revealed by 3D numerical modelling combined with LC-MS/MS. Water Res..

[2] Zhi Y., Zhao X., Qian S., Faria A.F., Lu X., Wang X., Li W., Han L., Tao Z., He Q. (2022). Removing emerging perfluoroalkyl ether acids and fluorotelomer sulfonates from water by nanofiltration membranes: Insights into performance and underlying mechanisms. Sep. Purifi. Technol..

[3] Zhou Z.G., Du H.-M., Dai Z., Mu Y., Tong L.L., Xing Q.J., Liu S.-S., Ao Z., Zou J.P. (2019). Degradation of organic pollutants by peroxymonosulfate activated by MnO_2_ with different crystalline structures: Catalytic performances and mechanisms. Chem. Eng. J..

[4] Ng L.Y., Mohammad A.W., Leo C.P., Hilal N. (2013). Polymeric membranes incorporated with metal/metal oxide nanoparticles: A comprehensive review. Desalination.

[5] Adityawarman D., Lugito G., Kawi S., Wenten I.G., Khoiruddin K. (2025). Advancements and future trends in nanostructured membrane technologies for seawater desalination. Desalination.

[6] Cheng S., Oatley D.L., Williams P.M., Wright C.J. (2012). Characterisation and application of a novel positively charged nanofiltration membrane for the treatment of textile industry wastewaters. Water Res..

[7] Bunani S., Yörükoğlu E., Sert G., Yüksel Ü., Yüksel M., Kabay N. (2013). Application of nanofiltration for reuse of municipal wastewater and quality analysis of product water. Desalination.

[8] Kang X., Ge Q. (2025). A critical review on the mechanism, progress and challenge of electrochemically assisted membrane cleaning in water treatment. Desalination.

[9] Casetta J., Ortiz D.G., Pochat-Bohatier C., Bechelany M., Miele P. (2023). Atomic layer deposition of TiO_2_ on porous polysulfone hollow fibers membranes for water treatment. Sep. Purifi. Technol..

[10] Li X., Nayak K., Stamm M., Tripathi B.P. (2021). Zwitterionic silica nanogel-modified polysulfone nanoporous membranes formed by in-situ method for water treatment. Chemosphere.

[11] Niestroj-Pahl R., te Brinke E., Roth H., Dähne L., de Vos W.M. (2025). Symmetric and asymmetric ceramic-supported polyelectrolyte multilayer nanofiltration membranes. J. Membr. Sci..

[12] Niu J., Yan T., Xiong Q., Lei Y., Zheng F., Patabendige A., Pan Z., Han G. (2025). Cation-modulated permselectivity regulation of polyelectrolyte nanofiltration membranes for water purification. Sep. Purifi. Technol..

[13] Cheng W., Liu C., Tong T., Epsztein R., Sun M., Verduzco R., Ma J., Elimelech M. (2018). Selective removal of divalent cations by polyelectrolyte multilayer nanofiltration membrane: Role of polyelectrolyte charge, ion size, and ionic strength. J. Membr. Sci..

[14] Feng X., Wang X., Lin X., Chen Y., Qi H. (2022). Mussel-inspired sulfated nanocellulose-mediated conductive nanofiber for thermoelectric and humidity sensing multifunctional applications. Chem. Eng. J..

[15] Dai F., Qian G., Ke Z., Xu K., Wang M., Li D., Deng Z., Yu Y., Chen C. (2024). Antifouling polyphenylene sulfone tight-ultrafiltration membrane by co-depositing dopamine and zwitterionic polymer for efficient dye/salt separation. Sep. Purifi. Technol..

[16] Lin G., Bai Z., Liu C., Liu S., Han M., Huang Y., Liu X. (2022). Mechanically robust, nonflammable and surface cross-linking composite membranes with high wettability for dendrite-proof and high-safety lithium-ion batteries. J. Membr. Sci..

[17] Gu G., Yang X., Li Y., Guo J., Huang J., Nxumalo E.N., Mamba B.B., Shao L. (2025). Advanced zwitterionic polymeric membranes for diverse applications beyond antifouling. Sep. Purifi. Technol..

[18] Liu H., Xie J., Zhao J., Wang R., Qi Y., Sun S. (2024). Temperature and photo sensitive PVDF-g-PNIPAAm/BN@PDA-Ag nanocomposite membranes with superior wasterwater separation and light-cleaning capabilities. Sep. Purifi. Technol..

[19] Wen Y., Wang J., Wang F., Wu H., Zhou J., Dai Z., Guo H. (2024). Recent advances in membranes modified with plant polyphenols in wastewater treatment: A review. Sep. Purifi. Technol..

[20] Pilařová V., Plachká K., Gazárková T., Švec F., Garrigues J.-C., Nováková L. (2025). Using artificial neural networks to elucidate retention interactions on stationary phases with amine moieties dedicated to supercritical fluid chromatography. Sep. Purifi. Technol..

[21] Li S., Zheng C., Tu L., Cai D., Huang Y., Gao C., Lu Y., Xue L. (2024). Construction of PDA-PEI/ZIF-L@PE tight ultra-filtration (TUF) membranes on porous polyethylene (PE) substrates for efficient dye/salt separation. J. Hazard. Mater..

[22] Jiang H., Yang N., Hao Y., Zhang L., Xiao X., Sun Y., Jiang B., Zhang L. (2024). Highly permeable polyamide nanofiltration membranes fabricated via the construction of anionic covalent organic frameworks/polydopamine composite interlayer. Sep. Purifi. Technol..

[23] Al Shaeli M., Orhun Teber O., Al Juboori R.A., Khataee A., Koyuncu I., Vatanpour V. (2024). Inorganic layered polymeric membranes: Highly-ordered porous ceramics for surface engineering of polymeric membranes. Sep. Purifi. Technol..

[24] Ma C., Chen C., Nikiforov A., Kajtazi A., An M., Gutierrez L., D’Haese A., Leus K., Van Der Voort P., Lynen F. (2024). Plasma-aerosol-assisted interface engineering of nanofiltration membranes to improve removal of organic pollutants from water. Chem. Eng. J..

[25] Choi Y.J., Saeed G., Lee D., Kwon S.H., Kim K.H. (2024). Enhanced electrochemical performance of flexible carbon substrates via carbonized layer of oxidant-induced polydopamine for high-performance supercapacitors. J. Ind. Eng. Chem..

[26] Zhu Z., Gao Q., Long Z., Huo Q., Ge Y., Vianney N., Daliko N.A., Meng Y., Qu J., Chen H. (2021). Polydopamine/poly(sulfobetaine methacrylate) Co-deposition coatings triggered by CuSO_4_/H_2_O_2_ on implants for improved surface hemocompatibility and antibacterial activity. Bioact. Mater..

[27] Zhang W., Liao Z., Meng X., Ai Niwaer A.E., Wang H., Li X., Liu D., Zuo F. (2020). Fast coating of hydrophobic upconversion nanoparticles by NaIO4-induced polymerization of dopamine: Positively charged surfaces and in situ deposition of Au nanoparticles. Appl. Surf. Sci..

[28] Chen Y., Liu Q. (2019). Oxidant-induced plant phenol surface chemistry for multifunctional coatings: Mechanism and potential applications. J. Membr. Sci..

[29] Huangfu C., Liu Z., Lu X., Liu Q., Wei T., Fan Z. (2021). Strong oxidation induced quinone-rich dopamine polymerization onto porous carbons as ultrahigh-capacity organic cathode for sodium-ion batteries. Energy Storage Mater..

[30] Bao Y., Xu W., Zhang J., Yang Q., Wang Z. (2024). Anti-fouling ampholytic polymer membrane with super-wetting surfaces for efficient oil-water emulsion separation. Desalination.

[31] Baig N., Salhi B., Ali S., Khan S.A., Mansha M., Khan N.A., Abdulazeez I., Kammakakam I. (2025). Synthesis of a novel polymer and design of carboxylate-terminated hyperbranched PEI-incorporated PVDF membranes for efficient oil-in-water emulsion separation. Sep. Purifi. Technol..

[32] Wang Z., Zhang W., Wen S., Wang L., Wang S., Wang Y., Lu J., Ma J., Cheng W. (2023). Rapid co-deposition of dopamine and polyethyleneimine triggered by CuSO_4_/H_2_O_2_ oxidation to fabricate nanofiltration membranes with high selectivity and antifouling ability. Sep. Purifi. Technol..

[33] Wu W., Wang Q., Li W., Liu W., Wang D., Fu J., Zhang J., Li Y., Wang H., Lu S. (2024). Investigation of the influence of amino trimethylene phosphonic acid on oxygen reduction reaction on platinum catalyst. Chem. Eng. J..

[34] Lv Y., Du Y., Chen Z.-X., Qiu W.-Z., Xu Z.-K. (2018). Nanocomposite membranes of polydopamine/electropositive nanoparticles/polyethyleneimine for nanofiltration. J. Membr. Sci..

[35] Chen G., Jin B., Zhao J., Li Y., He Y., Luo J. (2021). Efficient one-pot synthesis of mussel-inspired Cu-doped polydopamine nanoparticles with enhanced lubrication under heavy loads. Chem. Eng. J..

[36] Wang L., Fang F., Liu Y., Li J., Huang X. (2016). Facile preparation of heparinized polysulfone membrane assisted by polydopamine/polyethyleneimine co-deposition for simultaneous LDL selectivity and biocompatibility. Appl. Surf. Sci..

[37] Shi H., Xue L., Gao A., Fu Y., Zhou Q., Zhu L. (2016). Fouling-resistant and adhesion-resistant surface modification of dual layer PVDF hollow fiber membrane by dopamine and quaternary polyethyleneimine. J. Membr. Sci..

[38] Lv Q., Cai Y., Yang R., Zhang L., Han Y., Marfavi Z., Barazandeh M., Xu M., Zhang G., Zhang W. (2025). Efficient penetration and in situ polymerization of dopamine in biofilms for the eradication. Chem. Eng. J..

[39] Nie Z., Liu C., Jiang X., Zhou Y., Lin X., Zhao X., He Q., Chai H., Pang X., Ma J. (2022). Dopamine-triggered one-step codeposition of zwitterionic surfactants for anti-fouling polyethersulfone ultrafiltration membrane modification. Appl. Surf. Sci..

[40] Deng L., Li S., Qin Y., Zhang L., Chen H., Chang Z., Hu Y. (2021). Fabrication of antifouling thin-film composite nanofiltration membrane via surface grafting of polyethyleneimine followed by zwitterionic modification. J. Membr. Sci..

[41] Bera P., Trivedi J.S., Jewrajka S.K. (2023). Low fouling/scaling double network thin film composite nanofiltration membranes with improved desalination performance. Desalination.

[42] Jiang J., Zhu L., Zhu L., Zhu B., Xu Y. (2011). Surface Characteristics of a Self-Polymerized Dopamine Coating Deposited on Hydrophobic Polymer Films. Langmuir.

[43] Bandehali S., Parvizian F., Hosseini S.M., Matsuura T., Drioli E., Shen J., Moghadassi A., Adeleye A.S. (2021). Planning of smart gating membranes for water treatment. Chemosphere.

[44] Liu G., Matindi C.N., Pu Z., Kadanyo S., Cui Z., Yang J., Li J. (2024). Preparation of novel zwitterionic polysulfone ultrafiltration membranes for the dual-enhanced antifouling and antibacterial properties by in-situ one pot crosslinking reaction. J. Membr. Sci..

[45] Li Z., Hu K., Feng X. (2022). Co-depositing polyvinylamine and dopamine to enhance membrane performance for concentration of KAc solutions via sweeping air pervaporation. J. Membr. Sci..

[46] Chen L., Ren X., Li Y., Hu D., Feng X., Li W. (2022). Enhancing interface compatibility of UiO-66-NH_2_ and polyamide by incorporating dopamine into thin film nanocomposite membranes. J. Membr. Sci..

[47] Yang Y., Li Y., Goh K., Tan C.H., Wang R. (2022). Dopamine-intercalated polyelectrolyte multilayered nanofiltration membranes: Toward high permselectivity and ion-ion selectivity. J. Membr. Sci..

[48] Wang W., Zhang Y., Li F., Chen Y., Mojallali Rostami S.M., Hosseini S.S., Shao L. (2022). Mussel-inspired polyphenol/polyethyleneimine assembled membranes with highly positive charged surface for unprecedented high cation perm-selectivity. J. Membr.Sci..

[49] Lv Y., Yang H.-C., Liang H.-Q., Wan L.-S., Xu Z.-K. (2015). Nanofiltration membranes via co-deposition of polydopamine/polyethylenimine followed by cross-linking. J. Membr. Sci..

[50] Xiang J., Zhu R., Lang S., Yan H., Liu G., Peng B. (2021). Mussel-inspired immobilization of zwitterionic silver nanoparticles toward antibacterial cotton gauze for promoting wound healing. Chem. Eng. J..

[51] Ni X.X., Li J.H., Yu L.P. (2021). A novel PVDF hybrid membrane with excellent active–passive integrated antifouling and antibacterial properties based on a PDA guiding effect: Electronic supplementary information (ESI) available. Mater. Adv..

[52] Feng X., Peng D., Zhu J., Wang Y., Zhang Y. (2022). Recent advances of loose nanofiltration membranes for dye/salt separation. Sep. Purifi. Technol..

[53] Li Q., Sun W., Shang B., Zhang N., Zhao L., Deng H., Du P., Qiao M., Lu J., Wang Z. (2024). Lignin/mesoporous hollow silica nanosphere thin film nanocomposite loose nanofiltration membrane for dye/salt separation. Desalination.

[54] Ward L.M., Martin C.C., Weinman S.T. (2025). Pattern size relative to oil droplet size effect on oil fouling in nanofiltration. J. Membr. Sci..

[55] Qin Y., Qi P., Hao S., Shi W., Xiao J., Wang J., Hu Y. (2025). Methylation of reverse osmosis membrane for superior anti-fouling performance via blocking carboxyl groups in polyamide. Nat. Water.

[56] Zhao Y., Yang X., Cheng Z., Lau C.H., Ma J., Shao L. (2023). Surface manipulation for prevention of migratory viscous crude oil fouling in superhydrophilic membranes. Nat. Commun..

